# Gastric Antral Web in a 103-Year-Old Patient

**DOI:** 10.1155/2011/957060

**Published:** 2011-09-06

**Authors:** Waheed Gul, Khurram Abbass, Ronald J. Markert, Christopher J. Barde

**Affiliations:** ^1^Boonshoft School of Medicine, Wright State University, Dayton, OH 45435, USA; ^2^Dayton Veterans Affairs Medical Center, Dayton, OH 45428, USA

## 1. Introduction

Gastric antral web, also called antral diaphragm, is a rare cause of gastric-outlet obstruction. First described by Touroff et al. in 1940 [1], gastric antral web (GAW) or gastric antral diaphragm is a relatively rare and controversial entity. Both congenital and acquired etiologies have been postulated for this lesion in adults. The congenital theory recognizes that GAW occurs in infants and children. During the second month of embryologic development, the lumen of the developing digestive tract is plugged by a rapid overgrowth of epithelial cells. Vacuoles subsequently appear in the plugs and eventually coalesce to reestablish gut patency. The proposed mechanism for GAW is the failure of vacuoles to coalesce in the stomach [2]. However, acquired antral web in adults due to peptic diseases has also been documented [1, 3]. Approximately-one-quarter of all reported cases of GAW has been associated with either gastric or duodenal ulcer disease. Acquired etiology may be caused by scarring of the linear circumferential prepyloric and pyloric ulcers [4].

## 2. Case Report

**Figure 1:**
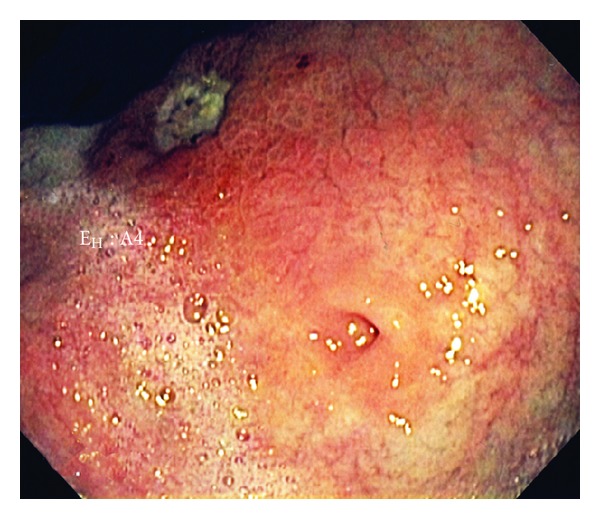
Endoscopic gastroduodenoscope showing gastric outlet obstruction with a very small opening in the antrum.

**Figure 2:**
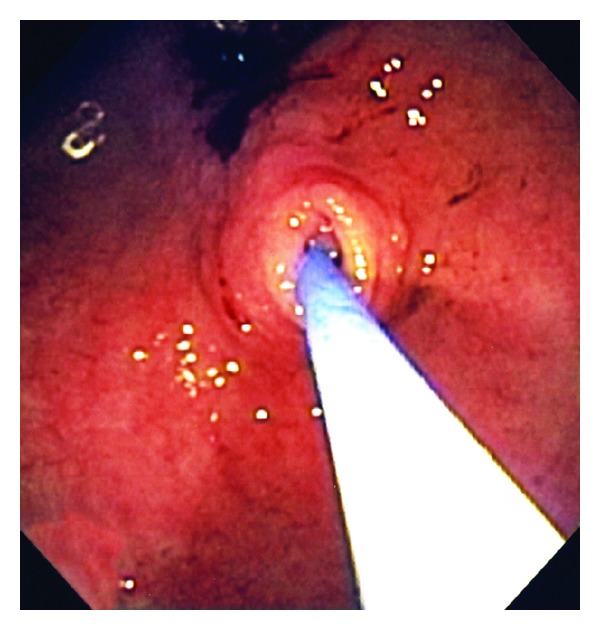
Endoscopic gastroduodenoscope showing opening in prepyloric web after probing with the tip of the heater probe.

**Figure 3:**
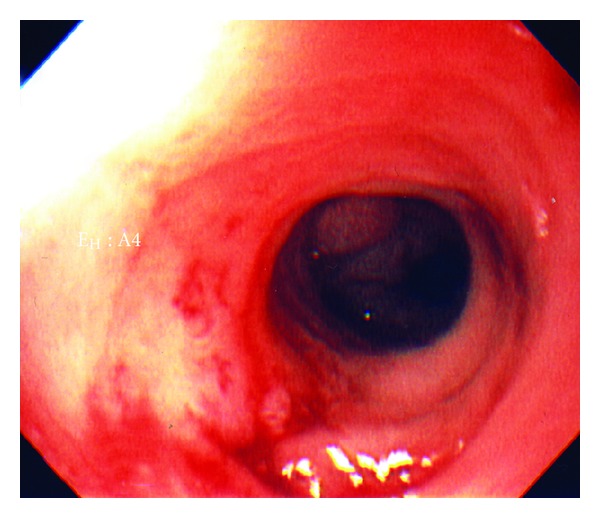
After-dilation, the pyloric channel was now patent. Endoscopic gastroduodenoscope passed easily up to the second part of the duodenum.

A 103-year-old female with a history of dementia, non-Hodgkin's lymphoma, and renal cell carcinoma, currently in remission, was admitted due to two episodes of hematemesis. The patient's family stated that she had a few months of nausea, vomiting, and decreased oral intake. Her hemoglobin was 7.8 g/dL, and her hematocrit was 22.4%. An endoscopic gastroduodenoscopy (EGD) showed a gastric ulcer at the incisura requiring local epinephrine and cauterization therapy. The EGD also showed gastric outlet obstruction with a very small opening in the antrum (Figure 1). After probing with the tip of the heater probe (Figure 2), we were able to pass through this opening and found that it was a prepyloric web. On a subsequent EGD, we dilated the prepyloric web with a Quantum TTC pyloric balloon dilator (Cook Medical, Bloomington, Ind, USA) using sizes 8 mm and 10 mm for one minute each. Post-dilation, the pyloric channel was patent (Figure 3), and we were able to pass the EGD scope easily up to the second part of the duodenum. On post dilation follow-up, the patient's nausea and vomiting resolved, and her oral intake improved.

## 3. Discussion

Infants with GAW present with persistent postprandial nonbile-stained vomiting and secondary failure to thrive [5]. In adults, the clinical presentation is variable. Symptoms depend on the size of the opening of the GAW. An opening larger than 1 cm causes no symptoms. Symptomatic adults present with postprandial fullness, epigastric pain, or both, and relief comes with vomiting [6]. With increasing age, symptoms have a late onset and may be due to ineffective mastication and progressive decrease in gastrointestinal tract motility. These problems prevent the stomach from pushing larger food boluses through the small aperture of the GAW [2]. We are not sure regarding exact etiology in our patient for the development of GAW but she possibly had the acquired antral web due to prepyloric ulcers.

The diagnosis of GAW is usually made by an upper gastrointestinal barium series or EGD. The classic feature is the double-bulb appearance: the normal duodenal bulb with a proximal antral chamber between the web and the pylorus [6]. EGD can show a large mucosal fold with a small aperture or a pinpoint pseuodpylorus [2, 4].

The management of GAW depends on symptoms and the size of the aperture. If the aperture is more than one cm and the patient is asymptomatic, no treatment is indicated other than dietary advice [4]. In contrast, symptomatic patients with a smaller aperture need either surgical or endoscopic intervention. Surgical options range from incision of the web with or without pyloroplasty to distal gastrectomy [7]. Endoscopic options include resection with a snare, papillotomy, or endoscopic Nd:YAG laser treatment [7]. We chose endoscopic pyloric balloon dilation due the patient's age and the family's wish for nonsurgical management.

## 4. Conclusion

We presented a case of GAW in a 103-year-old patient who was successfully managed with endoscopic pyloric balloon dilation. This treatment can be considered the therapy of choice due to its simplicity and low incidence of complications in elderly patients.

##  Disclaimer

The authors have no disclaimers to make regarding this publication.
